# Factors influencing hospitalized patients’ perception of individualized nursing care: a cross-sectional study

**DOI:** 10.1186/s12912-016-0137-7

**Published:** 2016-03-01

**Authors:** Stefan Köberich, Johanna Feuchtinger, Erik Farin

**Affiliations:** Institute for Quality Management and Social Medicine, Medical Center - University of Freiburg, Engelbergerstr. 21, 79106 Freiburg, Germany; Pflegedirektion, Heart Center – University of Freiburg, Hugstetter Str. 55, 79106 Freiburg, Germany; Quality and Development in Nursing Care, Medical Center - University of Freiburg, Breisacher Str. 62, Freiburg, Germany

**Keywords:** Patient-centered nursing care, Individualized care, Influencing factors, Germany, Hospital, Cross-sectional study

## Abstract

**Background:**

Individualized care is a cornerstone of patient-centered nursing care. To foster individualized care, influencing factors should be known. The aim of this study was to identify the individual and organizational factors influencing hospitalized patients’ perception of individualized care.

**Methods:**

A cross-sectional study was conducted of 606 patients from 20 wards from five hospitals across Germany. Individualized care and potential influencing factors were assessed via structured questionnaires. To identify influencing factors, we applied a hierarchical linear model with two levels.

**Results:**

Self-rated health, length of ward stay, educational level and shared decision-making process about nursing care were perceived to influence individualized care. A higher rating of health and longer ward stay correlated with improved perceptions of individualized nursing care. In addition, an educational level of nine or fewer years and a perceived shared decision-making process about nursing care positively influenced the perception of nursing care as being tailored to individual needs.

**Conclusions:**

Several factors influence patients’ perception of individualized care. However, only the decision-making process can be actively influenced by nurses. Therefore, nurses should be encouraged to promote shared decision-making regarding patients’ nursing care.

**Trial number:**

DRKS00005174 (Date of registration: 2013/08/01).

## Background

Patient-centered nursing care (PCNC), which puts the patient in the center of the care process and is holistic, individualized, tailored, respectful and empowering [1, 2], has been attracting attention for decades. The discussion in Germany about PCNC began in the late 1970s when nurses began to feel uncomfortable with the impersonal care of patients [3]. At that time, nursing care was based on Taylor’s principles, meaning that the head nurse had case responsibility for all patients, the head nurse assigned nursing tasks to staff members, and nursing tasks were usually executed in rounds [4]. Discussion about implementing a more patient-centered approach to nursing care led to changes in the system of delivering nursing care. Task-oriented system were shifted towards a patient-oriented one. Over the following decades, efforts were made to make nursing care more patient-centered via a model implementing a patient-centered nursing care delivery system, e.g. primary nursing [3].

The German healthcare system has undergone substantial changes since diagnosis-related groups (DRGs) were introduced in 2002. As a result, the number of hospitals and hospital beds fell; overall numbers of hospitalized patients and of care-dependent patients have risen, while the number of full-time nursing positions decreased [5]. Against this background, nurses fear that the shift in the nursing care delivery system towards a PCNC approach will be reversed [6, 7].

A growing number of studies have shown that PCNC is associated with improved patient outcomes. PCNC leads to better self-care [8, 9], makes patients more satisfied with care [8, 10, 11], and gives them a feeling of greater autonomy [12] and better quality of life [8, 12]. To introduce or maintain PCNC, or to adapt it to changing conditions, we need to know what influences patients’ perception of PCNC. Numerous studies have investigated the influence of patient- and organization-related variables on PCNC.

According to Suhonen and colleagues, age, gender, educational level, length of stay, and type of admission have a significant influence on how patients perceive care as individualized [13–15]. These results are in line with those evaluating influencing factors on patient satisfaction with care, which can be regarded as an outcome of individualized care [10, 16]. Higher age [17–20], gender (male) [18, 20–22], lower educational or socio-economic level [20–23] and better health status or quality of life [17, 24] are factors associated with better patient satisfaction with nursing care.

On the organizational level, the number of wards in a hospital and the number of beds per ward [25], as well as nurses’ work engagement and the ward’s service climate [26], seem to influence individualized care. In addition, surgical units [18, 21], primary nursing [27], better nurse-physician collaboration [24] and higher work engagement [17] predict higher satisfaction levels.

To the best of our knowledge, no study has investigated influencing factors on individualized nursing care in hospitals within the German healthcare setting. We therefore conducted a study aiming to explore factors that influence patients’ perception of individualized care. The following research question constituted the basis of the study: which individual and organizational factors influence patients’ perception of individualized nursing care?

## Method

### Design

This study’s design was that of a cross-sectional survey. Data were collected between October 2013 and July 2014 in five German hospitals.

### Study population

The study population comprised patients from 20 wards of five German tertiary care hospitals. Three of the five hospitals were run by church organizations and two were university-based public hospitals. Bed capacity in these hospitals ranged from 256 to 1395 beds. Patients were eligible to participate in this study if they remained at least three days on the participating wards and exhibited none of the following exclusion criteria: age <18 years; disorientation towards one of the following perspectives: time, person, situation or location; cognitive impairment or documented diagnosis of dementia; inability to fill out the questionnaire (in the study nurse’s view); lack of adequate fluency in the German language (reading comprehension); and inadequate vision.

### Sample size

Since consensus on the optimal sample size for a two-level hierarchical linear model does not exist, we applied as a rule-of-thumb 30 patients per ward, yielding for 20 wards a total of 600 patients [28].

### Data collection

Nursing directors of all hospitals intending to collect data about their nursing care delivery system with the “Instrument to Assess Nursing Care Delivery Systems” (“Instrument zur Erfassung von Pflegesystemen” (IzEP)) during our study period were asked to participate in this study. Information on which hospital was planning to evaluate its nursing care delivery system was provided by a member of the research group that developed IzEP (JF). If the nursing director was willing to participate, he or she signed a cooperation agreement to send the results from the nursing care system evaluation to our study group.

Eligible patients in the participating hospitals were asked to take part in this study by the local study coordinator. If patients expressed general willingness to participate, they were given written information on the study aims and procedure, the questionnaire, and a prepaid envelope. The patient was instructed to return the completed questionnaire in the sealed envelope to the local study coordinator, who posted the envelope to the principal investigator (SK), or to post it him/herself to the principal investigator. On receipt, the questionnaire was scanned and analyzed for missing data.

Voluntary consent was assumed if the patient returned the self-administered questionnaire. The questionnaire collected patients’ socio-demographic and disease-related data, as well as their perception of individualized care and the decision-making process about their nursing care.

### Measures

#### Individualized care scale

Patients’ perception of individualized nursing care was assessed via the German version of the Individualized Care Scale (ICS). The ICS is a questionnaire consisting of two scales (ICSA/ICSB) comprising 17 items each, which have to be answered on a five-point Likert scale ranging from 1 = “strongly disagree” to 5 = “strongly agree.” The ICSA assesses patients’ views on how individuality is supported through nursing interventions, while the ICSB assesses patients’ perceptions of individualized nursing care. ICSA and ICSB each contain three subscales labeled “clinical situation” (ClinA/ClinB), “personal life situation” (PersA/PersB), and “decisional control over care” (DecA/DecB).

The ICS was developed in the late 1990s [29, 30] and revised in 2005 [31] and 2010 [32]. The original version’s validity and reliability have been extensively investigated [29–32].

The ICS was translated into German in 2010, and its validity and reliability were initially assessed via a modified version in a psychiatric setting [33]. Its validity and reliability were established in an acute-care setting in 2014 [34]. Structural validity, known-group validity, and concurrent validity were assessed and approved as well. Cronbach’s alpha as a measure of reliability was revealed as excellent for the ICSA (α = 0.95; 95%CI: 0.94–0.95) and for the ICSB (α = 0.93; 95%CI: 0.92–0.94) [34].

#### Smoliner scale

We relied on the Smoliner Scale to assess the perceived decision-making process about nursing care. It is based on the framework of treatment decision-making devised by Charles and colleagues [35], who broke the treatment decision-making process down into three steps: (1) information exchange, (2) deliberation on received information, and (3) deciding on the treatment to implement. Smoliner and colleagues used this framework and translated it to the nursing context [36]. The Smoliner Scale consists of two subscales reflecting the decision-making process. The first asks about patients’ wishes regarding different steps in the decision-making process in nursing care, and the second assesses patients’ perception of the decision-making process. For the purpose of this study, we used the patients’ perception subscale that has three parts. The first part assesses patients’ perception about the information exchange and deliberation process using five statements answered on a six-point Likert scale ranging from 1 = “never” to 6 = “always.” The second part asks about their perception of personal involvement in the decision-making process in relation to various nursing tasks (e.g. hygiene, pain treatment) using a six-point Likert scale ranging from 1 = “never” to 6 = “always,” with an additional answer category (“not relevant”). The third part assesses patients’ perception of the type of decision-making process. To this end, the patient must indicate which of four statements best reflects the decision-making process. Two of the four statements can be assigned to a paternalistic decision-making process, one to a shared decision-making process, and one to an informed decision-making process. We only considered the third part of the patients’ perception subscale as a potential influencing factor on ICSA/ICSB. The first and second parts were excluded because they assessed factors similar to those in the ICSA and ICSB and would therefore correlate closely with them, as confirmed when the validity and reliability of the ICS were assessed [34]. The Smoliner Scale’s validity and reliability were assessed and revealed satisfactory psychometric properties. The Cronbach’s alpha of the subscale we used was 0.86.

#### Instrument to assess nursing care delivery systems (IzEP)

To assess the nursing care delivery system of participating wards, we used the Instrument to Assess Nursing Care Delivery Systems (IzEP) [37]. IzEP is a multidimensional instrument consisting of nine sub-questionnaires that address five aspects of a nursing care delivery system. To assess these aspects, head and staff nurses, patients, relatives, therapists, physicians, and external contacts had to fill out one of the questionnaires. Patient records and duty rosters were also analyzed. Results of the questionnaires were triangulated and merged to an overall score ranging from zero to 100. A sum-score between zero and 10 indicated that no specific system existed on a ward. A sum-score of 11 to 40 reflected a task-oriented nursing care model. A sum-score of 41 to 75 indicated a zone nursing model, and a sum-score above 75 reflected a patient-oriented care model. In the task-oriented nursing care model, the head nurse has case responsibility for all patients and nursing tasks are assigned by the head nurse to staff members. In the zone nursing care model, the head nurse also has case responsibility, but nurses are responsible for a group of patients for a limited time (e.g. one shift). In the patient-oriented care model (e.g. primary nursing), case responsibility is decentralized, and a dedicated nurse (primary nurse) assumes case responsibility for one or more patients during their entire stay.

The IzEP’s psychometric properties were assessed and confirmed [37]. Inter-rater reliability and test-retest reliability of the instrument were assessed and rated as good and very good, with AC1 ranging from 0.61 to 1.0. Validity was confirmed by experts’ rating of the nursing care delivery system compared with the IzEP-assessed nursing care delivery system. In addition, the unidimensionality of the instrument section was approved by confirmatory factor analysis (unpublished data).

#### Socio-demographic and health-related variables

Socio-demographic and health-related variables were collected with an additional questionnaire. Patients were asked to state their age, gender, nationality, marital status, educational status, if their hospital stay was planned or unplanned, why they were being hospitalized, how long their stay was on the ward, and how they rated their perceived health. Perceived health was rated on a six-point Likert scale where 1 is excellent and 6 is very poor.

### Ethical considerations

Our data protection protocol was approved by the data protection officer of the Medical Center – University of Freiburg, Germany. Our study protocol was approved by the Ethics Committee of Albert-Ludwigs-University of Freiburg, Germany (EK-Freiburg 318/13). The study complied with the principles outlined in the Declaration of Helsinki and was registered in the German Clinical Trials Register (DRKS00005174).

### Data analysis

Data were coded and entered into IBM SPSS Version 22. To analyze the influence of variables on the patient and ward level on perceived individualized care, we applied a hierarchical linear model with two levels (two-level random coefficient model). Calculations were made with HLM 7 (Scientific Software International, Lincolnwood, IL, USA).

Missing data were handled as follows: patient data were excluded from the analysis (listwise deletion) if more than 20 % of items were missing on any one of three scales (ICSA, ICSB, or the ‘Experience’ subscale of the Smoliner scale); otherwise pairwise deletion was used. To describe patients’ socio-demographics and disease-specific characteristics, we used descriptive statistics. Nominally-scaled variables are displayed as numbers and percentages, interval-scaled, normally distributed variables as mean and standard deviation (SD). If interval-scaled variables are not distributed normally, they are displayed as medians and interquartile ranges (IQR).

In the first step, patient and organizational variables were correlated with the sum-score of ICSA and ISCB, respectively. Personal variables were: age, gender, nationality, marital status, educational level, planned/unplanned hospital stay, perceived decision-making process (Smoliner Scale), self-rated health, length of stay. Organizational variables included in the bivariate analyses were: number of beds per ward, number of full-time nursing positions per ward, total number of registered nurses per ward, and the ward’s nurse: bed ratio, calculated by dividing the number of beds by the number of full-time nursing positions per ward, nursing-bedside handover, and the nursing care delivery system (IzEP). We created dummy variables for the categorical variables.

If the correlation’s *p*-value between influencing variables and ICSA/ICSB was ≤ 0.20, the variables were entered into the two-linear hierarchical model. This was done to reduce multicollinearity and produce parsimonious models. Further variables were deleted if multicollinearity was detected by the HLM7 software. In that case, multicollinearity analyses were conducted with IBM SPSS and variables with the highest variance inflation factor-value were deleted.

To evaluate the data’s hierarchical structure, we calculated intraclass correlations (ICC). If the ICC approached zero, then the grouping by wards was of no use, as there was no variance to explain on the ward level.

Because only 12 out of 20 wards provided us with data about their nursing care systems, we decided to carry out two analyses. In the first, we included IzEP, in the second we excluded it. We therefore calculated four models in all (influencing factors on ICSA/ICSB with/with no data on nursing care delivery system assessed with IzEP).

## Results

A total of 884 patient questionnaires were distributed, of which 699 (79.1 %) were returned. Of those, 93 (13.3 %) were excluded because of missing data, leaving 606 questionnaires for data analysis.

### Patient characteristics

The participants were predominantly male, with a median age of 59 years, and mostly German, with education of up to nine years. Planned and unplanned hospital stays were nearly equal in number, and the median length of hospital stay was eight days. Health was perceived as satisfactory (Table 1).

**Table 1:** Participant profile

	All (*n* = 606)
	*n* (%)
Gender	
Female	244 (40.3)
Male	360 (59.4)
Age (years)^b^	59 (48;70)
Nationality	
German	579 (95.5)
Other	22 (3.6)
Marital status	
Single	102 (16.8)
Married	376 (62.0)
Divorced/living apart	71 (11.7)
Widowed	54 (8.9)
Educational level	
≤ 9 years	247 (40.8)
10 years	180 (29.7)
13 years	98 (16.2)
13 years + university degree	72 (11.9)
Hospital stay was	
Planned	318 (52.5)
Unplanned (e.g. emergency admission)	277 (45.7)
Length of hospital stay (days)^b^	8 (5;11)
Self-rated health^a,b^	3 (2;3)

NOTE: ^b^Median (Interquartile range); ^a^Likert scale response pattern (1 = very good; 6 = very poor)

### Ward characteristics

Characteristics of the participating wards are displayed in Table 2. The medical disciplines of the wards are manifold, with cardiology being the most frequent (*n* = 4). The median number of beds was 27.5, ranging from 16 to 40 beds. The mean number of full-time positions on a ward was 12.9, yielding an average nurse-bed ratio of 1:2.2. Twelve of 20 wards provided data about their nursing care system: one ward (8.3 %) had a task-oriented nursing care system, two wards (16.6 %) had a patient-oriented nursing care system, and nine wards (75.0 %) had a zone nursing system. Seventy percent of the wards (*n* = 14) conducted their nursing handover at the bedside.

**Table 2:** Ward profile

Wards	Discipline	Number of beds	Number of FTP^a^	Nurse: bed ratio	Nursing care delivery system	Bedside Nursing-handover	ICSA	ICSB
Hospital A							3.73 (±0.89)	4.14 (±0.71)
Ward A	Orthopedics	36	11.0	1 : 3.27	Patient-oriented	Yes	3.75 (±0.90)	4.13 (±0.71)
Ward B	Orthopedics	22	8.8	1 : 2.50	Patient-oriented	Yes	3.98 (±0.71)	4.38 (±0.47)
Ward C	Gastroenterology	38	14.1	1 : 2.70	n.a.	Yes	3.84 (±0.91)	4.28 (±0.68)
Ward D	Cardiology	38	15.4	1 : 2.47	n.a.	Yes	3.28 (±1.01)	3.74 (±0.87)
Ward E	General surgery	38	17.2	1 : 2.21	n.a.	Yes	3.72 (±0.81)	4.15 (±0.64)
Hospital B							3.46 (±0.96)	3.90 (±0.71)
Ward A	General surgery	32	10.59	1 : 3.02	Zone nursing	Yes	3.73 (±1.14)	4.02 (±0.88)
Ward B	Neurology	30	19.0	1 : 1.58	Zone nursing	Yes	3.16 (±1.09)	3.71 (±0.96)
Ward C	Mixed (urology/general surgery)	26	9.16	1 : 2.84	Zone nursing	Yes	3.42 (±0.84)	3.89 (±0.68)
Ward D	Mixed (urology/general surgery)	25	11.15	1 : 2.24	Zone nursing	Yes	3.55 (±0.71)	4.01 (±0.53)
Hospital C							3.82 (±0.86)	4.30 (±0.64)
Ward A	Mixed (gynecology/orthopedics)	30	12.56	1 : 2.39	n.a.	No	4.03 (±0.95)	4.55 (±0.58)
Ward B	Ear, nose and throat	37	9.20	1 : 4.02	Task-oriented	No	3.66 (±0.90)	4.15 (±0.70)
Ward C	Urology	40	13.93	1 : 2.87	n.a.	No	3.91 (±0.65)	4.29 (±0.51)
Ward D	Traumatology	29	13.11	1 : 2.21	n.a.	No	3.64 (±0.91)	4.17 (±0.71)
Hospital D							3.66 (±0.93)	4.10 (±0.66)
Ward A	Cardiology	16	11.35	1 : 1.41	Zone nursing	Yes	3.80 (±0.77)	4.25 (±0.54)
Ward B	Cardiology	23	15.00	1 : 1.47	Zone nursing	Yes	3.74 (±1.10)	4.22 (±0.70)
Ward C	Cardiology	16	11.45	1 : 1.40	Zone nursing	Yes	3.42 (±1.08)	4.01 (±0.76)
Ward D	Heart surgery	22	16.00	1 : 1.38	Zone nursing	Yes	3.81 (±0.77)	4.03 (±0.66)
Ward E	Heart surgery	21	16.00	1 : 1.31	Zone nursing	Yes	3.56 (±0.84)	3.98 (±0.58)
Hospital E							3.59 (±0.83)	4.02 (±0.61)
Ward A	Dermatology	24	11.61	1 : 2.06	n.a	No	3.61 (±0.81)	4.05 (±0.73)
Ward B	Dermatology	24	10.50	1 : 2.29	n.a	No	3.58 (±0.86)	3.99 (±0.50)

NOTE: *FTP* full-time positions, *n.a.* not assessed; ^a^occupied at data collection time; *ICSA* part A of the individualized care scale, *ICSB* part B of the individualized care scale

### Bivariate correlations

For six organizational variables (OV) and nine individual variables (IV), the *p*-value of the correlation between ICSA and individual/organizational variable was ≤0.20. For the ISCB 5 OV and 7 IV reached the threshold of *p* ≤ 0.2. After we checked for multicollinearity, 4 OV and 8 IV (ICSA) and 3 OV and 7 IV (ICSB) could be included in the multi-level analysis (Table 3).

**Table 3:** Hierarchical linear model variables

	ICSA	ICSB
Level “Ward”	Bedside handover	Bedside handover
	Number of full-time positions	Number of full-time positions
	Patient-oriented nursing care	
	Percentage of registered nurses	Percentage of registered nurses
Level “Patient”	Education ≤ 9 years	Education ≤ 9 years
	Education ≥ 12 years	Education ≥ 12 years
	Length of stay	Gender
	Marital status: divorced/living apart	Length of stay
	Marital status: married	Perceived decision-making process: paternalistic
	Perceived decision-making process: informed	Perceived decision-making process: shared
	Perceived decision-making process: shared	Self-rated health
	Self-rated health	

Variables which correlated with the target variable (ISCA/ISCB) on a p-level above 0.2 or which were excluded because of multicollinearity from the analysis of the two-level hierarchal model are displayed in Table 4.

**Table 4:** Variables excluded from hierarchical linear model

	ICSA	ICSB
Level “Ward”	Number of beds	Number of beds
	Ratio registered nurse:number of beds	Ratio registered nurse:number of beds
	Task-oriented nursing care	Task-oriented nursing care
	Zone nursing care	Zone nursing care
		Patient-oriented nursing care
Level “Patient”	Age	Age
	Education = 10 years	Education = 10 years
	Gender	Type of hospital admission
	Type of hospital admission	Marital status: divorced/living apart
	Marital status: single	Marital status: married
	Marital status: widowed	Marital status: single
	Nationality	Marital status: widowed
	Perceived decision-making process: paternalistic	Nationality
		Perceived decision-making process: informed

### Hierarchical structure of data

For the ICSA with and with no data on the nursing care delivery system, ICC was 0.186 (*p* = 0.018) and 0.189 (*p* = 0.002), respectively, indicating that about 19 % of the total individual differences in ICSA occurred on the ward level.

For the ICSB with data on the nursing care delivery system, ICC was 0.187 (*p* = 0.015) and without data on the nursing care delivery system, ICC was 0.189 (*p* = 0.001).

### Influencing factors

#### With IzEP data

To assess factors influencing patients’ views of how individuality is supported through nursing interventions (ICSA), we included 268 cases in the analysis. On the ward level, we detected no statistically significant variables that influenced patients’ views, but on the patient level, self-rated health, educational level, and perceived decision-making about nursing interventions influenced the ICSA sum-score statistically significantly. Better self-rated health (*γ* = -0.149; *p* = 0.027), an educational level of ≤ 9 years (*γ* = 0.285; *p* = 0.042) and a decision-making process perceived as shared (*γ* = 0.478; *p* < 0.001) were associated with higher ICSA scores (Table 5).

**Table 5:** Hierarchical linear model levels for ICSA

	ICSA with IzEP (*n* = 268)			ICSA without IzEP (*n* = 455)		
Variables	Coefficient	Standard error	*p*-value	Coefficient	Standard error	*p*-value
For INTRCPT1, *β*_*0*_						
INTRCPT2, *γ*_*00*_	3.581651	0.051688	<0.001	3.634260	0.039962	<0.001
FTP, *γ*_*01*_	-0.023683	0.018320	0.197	-0.018002	0.013996	0.199
RN, *γ*_*02*_	-0.011271	0.010031	0.262	-0.004339	0.006252	0.488
PONC, *γ*_*03*_	0.209146	0.180333	0.247	**-**	**-**	**-**
BSH, *γ*_*04*_	-0.036413	0.229578	0.874	-0.087099	0.094867	0.359
For LENGTH slope, *β*_*1*_						
INTRCPT2, *γ*_*10*_	0.008225	0.007173	0.253	0.012785	0.004168	0.002
For HEALTH slope, *β*_*2*_						
INTRCPT2, *γ*_*20*_	-0.148911	0.066762	0.027	-0.166410	0.046058	<0.001
For MS-M slope, *β*_*3*_						
INTRCPT2, *γ*_*30*_	-0.022301	0.122917	0.856	0.095603	0.093973	0.310
For MS-D slope, *β*_*4*_						
INTRCPT2, *γ*_*40*_	-0.183245	0.180046	0.310	-0.098279	0.137446	0.475
For EDU9 slope, *β*_*5*_						
INTRCPT2, *γ*_*50*_	0.284946	0.139644	0.042	0.057950	0.096949	0.550
For EDU12 slope, *β*_*6*_						
INTRCPT2, *γ*_*60*_	0.028198	0.135264	0.835	-0.092431	0.105262	0.380
For SHARED slope, *β*_*7*_						
INTRCPT2, *γ*_*80*_	0.477949	0.106675	<0.001	0.401915	0.086817	<0.001
For INFORMED slope, *β*_*8*_						
INTRCPT2, *γ*_*80*_	-0.200338	0.167714	0.233	-0.185940	0.129215	0.151

NOTE: *INTRCPT* intercept, *FTP* full-time positions, *RN* percentage of registered nurses, *PONC* patient-oriented nursing care, *BSH* bedside handover, *LENGTH* length of stay, *HEALTH* self-rated health, *MS-M* marital status – married, *MS-D* marital status – divorced, *EDU9* education ≤ 9 years, *EDU12* education > 12 years, *SHARED* perceived shared decision-making, *INFORMED* perceived informed decision-making

For the ICSB, only self-rated health (*γ* = -0.121; *p* = 0.018) and shared decision-making (*γ* = 0.445; *p* < 0.001) influenced the perceived individualization of nursing care. This analysis included 267 cases (Table 6).

**Table 6:** Hierarchical linear model levels for ICSB

	ICSB with IzEP (*n* = 267)			ICSB without IzEP (*n* = 456)		
Variables	Coefficient	Standard error	*p*-value	Coefficient	Standard error	*p*-value
For INTRCPT1, *β*_*0*_						
INTRCPT2, *γ*_*00*_	4.045164	0.040483	<0.001	4.098297	0.030334	<0.001
FTP, *γ*_*01*_	-0.008109	0.009385	0.388	-0.017996	0.011392	0.115
RN, *γ*_*02*_	-	-	-	-0.004747	0.004753	0.318
PONC, *γ*_*03*_	0.108555	0.114423	0.344	-	-	
BSH, *γ*_*04*_	0.012850	0.175160	0.942	-0.114752	0.075144	0.127
For LENGTH slope, *β*_*1*_						
INTRCPT2, *γ*_*10*_	0.007694	0.006873	0.264	0.009698	0.003816	0.011
For HEALTH slope, *β*_*2*_						
INTRCPT2, *γ*_*20*_	-0.121351	0.051178	0.018	-0.145374	0.036024	<0.001
For GEN slope, *β*_*3*_						
INTRCPT2, *γ*_*30*_	0.073777	0.089974	0.413	0.069189	0.065792	0.294
For EDU9 slope, *β*_*4*_						
INTRCPT2, *γ*_*40*_	0.142473	0.109294	0.194	0.087852	0.074109	0.236
For EDU12 slope, *β*_*5*_						
INTRCPT2, *γ*_*50*_	0.060791	0.108330	0.575	-0.031406	0.080597	0.697
For PAT slope, *β*_*6*_						
INTRCPT2, *γ*_*60*_	0.102604	0.129714	0.430	0.059263	0.094503	0.531
For SHARED slope, *β*_*7*_						
INTRCPT2, *γ*_*70*_	0.444839	0.121533	<0.001	0.385917	0.088600	<0.001

NOTE: *INTRCPT* intercept, *FTP* full-time positions, *RN* percentage of registered nurses, *PONC* patient-oriented nursing care, *BSH* bedside handover, *LENGTH* length of stay, *HEALTH* self-rated health, *GEN* gender, *EDU9* education ≤ 9 years, *EDU12* education > 12 years, *PAT* perceived paternalistic decision-making, *SHARED* perceived shared decision-making

#### Without IzEP data

Assessing influencing factors on ICSA and ICSB excluding data on the nursing care delivery system, we included 455 (ICSA) and 456 (ICSB) cases in the analysis. For both scales, length of stay, self-rated health and shared decision-making influenced the perceived individualization of nursing care. A longer stay (ICSA: *γ* = 0.013, *p* = 0.002; ICSB: *γ* = 0.010, *p* = 0.011), better perceived health (ICSA: *γ* = -0.166, *p* < 0.001; ICSB: *γ* = -0.145, *p* < 0.001) and a decision-making process perceived as shared (ICSA: *γ* = 0.402, *p* < 0.001; ICSB: *γ* = 0.386, *p* < 0.001) influenced the perception of individualized nursing care positively (Tables 5 and 6).

## Discussion

The results of our study suggest that educational level, length of hospital stay, self-rated health, and the perceived decision-making process influence patients’ perception of individualized care. In detail: the longer the patient remains in hospital and the better the patient rates his/her health, the more those patients perceive the nursing care as being tailored to their individual needs and wishes. In addition, an education lasting nine years or less and perceiving the decision-making process in nursing care as being shared are associated with perceiving care as more individualized.

Patients’ educational level has frequently been described as an influencing factor on perceived patient-centered nursing care. Suhonen and colleagues observed the trend whereby orthopedic and trauma patients from five different countries (Finland, Greece, Sweden, the UK and the USA) perceived their care as less individualized the higher their educational level was [14]. This is in line with other studies using the ICS [13, 15]. Radwin [38] observed the same trend in oncology patients. Results of studies which evaluated influencing factors on patients’ satisfaction with nursing care also suggest an association between a patient’s educational level and the level of satisfaction [20–23]. In a systematic review about patient satisfaction with nursing care, Johansson and colleagues [39] suggest that the higher patients’ educational level is, the higher their expectations of nursing care. Patients with a higher educational level may have higher expectations regarding the information they are given and their overall care. If these expectations are not met, patients rate their satisfaction with nursing care as low. Results from a systematic review revealed that patients of higher socio-economic status (including their educational level [40]) communicated more actively and elicited more information from their doctors than those of lower socio-economic status. One can assume that patients are more likely to be disappointed with treatment results when the information they wanted has not been provided. To the best of our knowledge, there is a lack of research regarding communication style, desired information, and the decision-making process in nursing care depending on patients’ educational level which may help to add evidence for the aforementioned assumption.

In our study, patients with a longer ward stay perceived their nursing care as more individualized than those whose stay was shorter. This result is also in line with findings from a study by Land and Suhonen [13]. Charalambous [41] assessed cancer patients’ satisfaction with nursing care and its relation to different variables. Among others, length of stay had a significant influence on patients’ satisfaction with care. The longer the stay, the more satisfied they were with their nursing care. Charalambous suggests that patients discharged early worry about the continuity of their care or lack information on self-care activities at home, all of which lead to a lower level of satisfaction. We hypothesize that patients who stay longer on a specific ward may develop a stronger relationship with the nurses and are thus able to communicate their wishes and needs more freely, and that nurses are better able to consider those wishes and needs. However, more studies exploring the relationship between length of stay and perceived patient-centeredness in nursing care are necessary.

Self-rated health exerts an influence on the perceived individualization of nursing care. The better a patient rates his or her health, the more nursing care is considered to be patient-centered. Results from a study by Suhonen and colleagues [15] with 861 patients from six hospitals all over Finland suggest a positive relationship between quality of life and the perceived individualization of nursing care. The authors hypothesize that patients with better self-rated health have fewer care demands (that are more easily fulfilled) than those with worse self-rated health.

Higher age has been described in numerous studies as a factor associated with higher perceived individualized care [14, 15, 30]. It is suggested that older patients are more tolerant, less demanding, more respectful of professional authorities [42], do not complain easily or do not tell healthcare providers their wishes and thoughts [15]. However, a consistent and clear explanation for this association has not been provided so far. Interestingly, our results do not confirm this association, and are in line with the results of a study conducted by Land and Suhonen [13]. We have no explanation for our results, and therefore we hope that further studies with a mixed-method approach will help us discover whether there is an association between age and perceived individualized care.

The same applies to our results regarding gender. We identified no association between gender and perceived individualized care, although some suggest that female patients perceive their care differently [43] in terms of being less satisfied with the care they receive, which is line with findings from other studies [13, 14, 22]. As there is no evidence-based explanation, we can only suggest that our cohort is quite homogeneous in their attitude towards and experience of nursing care and that therefore patients of different age and different gender perceive their care similarly.

A new finding from our study is that perceived individualized nursing care is positively associated with perceived shared decision-making. Patients perceiving the decision-making process as being shared experienced their care as more tailored to their specific needs and wishes than those who experienced the decision-making process as paternalistic or informed. Although Suhonen and colleagues [25] discovered that individualized care is stimulated by patient-centered nurse-patient interaction (i.e. primary nursing), to the best of our knowledge no study has evaluated the relation between different approaches in the decision-making process and the individualization of nursing care. On the other hand, considering the examination of patient-centered care concept by Kitson and colleagues [44], our study results seem plausible. Kitson and colleagues identified common elements of patient-centered care in health policy, medical and nursing literature, and developed three key aspects of patient-centered care: (1) patient participation and involvement, (2) the relationship between the patient and the health professional, and (3) the context in which care is delivered. Sub-themes of the first core element reflect the individualized approach to care. The themes Kitson and colleagues identified are: (1) patient participation as a respected and autonomous individual; (2) a care plan based on the patient’s individual needs; and (3) addressing patient’s physical and emotional needs. All three subthemes reflect elements of individualized nursing care according to the definition by Suhonen and colleagues [29–31].

Some authors suggest that bedside nursing handover influences how individualized patients perceive their care [7, 45]. However, although the bivariate correlation between bedside handover and individualized care suggests a positive relationship in our study, in the two-level hierarchical model, nursing bedside handover revealed no influence on the perceived patient-centeredness of nursing care. There seem to be other interventions exerting a greater influence on shared decision-making and therefore on perceived individualized care.

### Study limitations

This study has several limitations. Cross-sectional studies provide only a snapshot of an actual situation. Therefore, it is possible that our results would differ had another time-frame of data collection been chosen. It is thus difficult to make any causal inferences.

In addition, this study was conducted within the German health care system. Therefore, results need to be interpreted in the context of the German healthcare setting. Our results cannot be generalized. Further studies are needed to replicate these study results in countries with different health care systems. Furthermore, more studies are needed within the German health care setting to replicate our results using different hospitals.

### Strengths

To assess influencing factors on perceived individualized care of hospitalized patients, we conducted a cross-sectional study and analyzed data using a two-level hierarchical linear model. Multilevel models take into account that data are clustered in groups that tend to respond similarly. In applying such models we were able to separate the influence of ward and patient on perceived individualized care.

### Implications

As the decision-making process is the only variable that can be actively influenced by nurses, efforts to involve patients in decision-making about their care should be encouraged. Promoting shared decision-making in nursing care should become a priority in nursing education.

## Conclusion

Patients’ perception of individualized nursing care is influenced by the length of hospital stay, patients’ self-rated health, patients’ educational level, and patients’ perception of shared decision-making within the nursing care process. A longer hospital stay, better perceived health, a lower educational level and experienced shared decision-making is associated with a perception of more individualized care.

## Abbreviations

ClinA/ClinB: Subscale “clinical situation” of Individualized care scale – Part A/B
DecA/DecB: subscale “decisional control over care” of individualized care scale – Part A/B
PCNC: patient-centered nursing care
ICC: intraclass correlation coefficient
ICS: individualized care scale
ICSA: individualized care scale - Part A
ICSB: individualized care scale – Part B
IV: individual variables
IzEP: Instrument zur Erfassung von Pflegesystemen (Instrument to Assess Nursing Care Delivery Systems)
OV: organizational variables
PersA/PersB: subscale “personal life situation” of individualized care scale – Part A/B
